# Identification of factors associated with failure to meet nocturnal penile tumescence and rigidity criteria based on RigiScan monitoring and machine learning algorithms: a cross-sectional study

**DOI:** 10.3389/fendo.2026.1926525

**Published:** 2026-10-27

**Authors:** Jian Wang, Yang Shan, Silun Li, Zihao Sun, Zhenrong Piao, Xiaoyan Zhang, Maosen Zhang

**Affiliations:** 1 Department of Andrology, Yangzhou Hospital of Traditional Chinese Medicine, Yangzhou, China; 2 Department of General Surgery, Affiliated Hospital of Nanjing University of Chinese Medicine/Jiangsu Province Hospital of Chinese Medicine, Nanjing, China; 3 Department of Respiratory Medicine, Sichuan First Hospital of Traditional Chinese Medicine, Chengdu, China; 4 Department of Andrology, Affiliated Hospital of Nanjing University of Chinese Medicine/Jiangsu Province Hospital of Chinese Medicine, Nanjing, China; 5 School of Medicine, Nanjing University of Chinese Medicine, Nanjing, China

**Keywords:** machine learning, nocturnal penile tumescence and rigidity, organic erectile dysfunction, RigiScan, SHAP

## Abstract

**Background:**

Using RigiScan nocturnal penile tumescence and rigidity (NPTR) monitoring as reference, we applied machine learning to identify routine clinical indicators associated with failure to meet NPTR criteria (no effective nocturnal erection).

**Methods:**

A total of 519 patients with erectile dysfunction completed two consecutive nights of RigiScan monitoring and were divided into effective and no-effective nocturnal erection groups. Data were split 7:3 into training (n = 364) and internal validation (n = 155) sets. Feature selection (univariate logistic regression, Boruta, LASSO) was performed exclusively in the training set. Five models (LR, RF, SVM, LightGBM, XGBoost) were constructed. The classification threshold was determined in the training set and fixed in the validation set. Performance was assessed using AUC with bootstrap 95% CIs, Brier score, calibration slope and intercept, sensitivity, specificity, PPV, and NPV. Nested cross-validation (5 × 3) was performed. SHAP interpreted the best model.

**Results:**

The effective group comprised 389 patients and the no-effective group 130. Six variables were selected: HbA1c, GLU, Smoke, Hypertension, Age, and BMI. In the validation set, LR achieved the highest AUC (0.779, 95% CI 0.692–0.861), followed by LGBM (0.718), XGB (0.700), RF (0.699), and SVC (0.646). DeLong’s test showed LR significantly outperformed RF (p = 0.028) and SVC (p = 0.009), but not LGBM or XGB. In nested cross-validation, LR also had a higher mean AUC than RF (0.726 ± 0.052 vs 0.666 ± 0.034). LR showed the lowest Brier score (0.151) and calibration slope closest to 1 (1.258). SHAP identified BMI, GLU, and Age as the top three contributors.

**Conclusions:**

Conventional metabolic indicators achieved only moderate discrimination. BMI, GLU, and HbA1c were consistently associated with failure to meet NPTR criteria. LR was the most robust model but cannot replace RigiScan monitoring. These findings should be interpreted as associations, not causal relationships.

## 1 Introduction

Erectile dysfunction (ED) is defined as the persistent inability to achieve or maintain an erection sufficient for satisfactory sexual performance (1). With the accelerating pace of modern life, the incidence of ED has shown a trend toward younger age groups, and it has become one of the common male disorders (2, 3). Etiologically, ED can be classified into organic, psychogenic, and mixed types. Organic ED is often secondary to systemic diseases such as diabetes mellitus and cardiovascular disease, with the core pathological mechanisms involving neurovascular damage and corporal fibrosis (4); in addition, iatrogenic factors such as medications (e.g., 5α-reductase inhibitors, neuropsychiatric drugs) and urological surgeries also account for a certain proportion (5–7). Psychogenic ED, on the other hand, is mostly associated with psychological states such as stress, anxiety, and depression, which can disrupt the neurochemical pathways of sexual arousal (5). In clinical practice, however, purely psychogenic or purely organic ED is relatively uncommon, and most patients present with mixed ED; therefore, the terms “primarily organic” or “primarily psychogenic” may be more clinically appropriate (5). ED not only imposes a psychological burden on patients, inducing anxiety and depression, but may also impair family relationships and social functioning; long-term lack of intervention can reduce self-identity, adversely affect partner relationships and social support, and severely diminish quality of life. Moreover, ED is now regarded as an early warning signal of cardiovascular events, and its presence can increase the risk of myocardial infarction and all-cause mortality (5, 8, 9). Consequently, strengthening the prevention, early identification, and comprehensive management of ED is of considerable clinical importance.

Currently, nocturnal penile tumescence and rigidity (NPTR) monitoring is widely used to differentiate psychogenic from organic ED. However, its interpretation depends on sleep, age, depression, and other factors, and failure to meet the NPTR efficacy criterion does not equate to independently confirmed organic ED (10–12). The RigiScan device, with its high sensitivity and reproducibility, has become the most widely used standardized instrument, and its interpretation criteria (tip rigidity ≥ 60% and effective erection duration ≥ 10 min) have been adopted by national and international guidelines. If NPTR results meet the efficacy criterion, organic pathology is unlikely; if abnormal, an organic cause is more likely. However, RigiScan testing is limited by equipment cost, operational duration, and environmental requirements, making it difficult to implement as a routine screening tool. To our knowledge, the investigation of factors associated with failure to meet NPTR criteria based on objective RigiScan grading remains insufficient. Although conventional factors such as age and metabolic diseases are known to be associated with ED, their relative contributions and potential nonlinear associations with objective NPTR outcomes remain unclear. Traditional regression models struggle to capture complex patterns in high-dimensional data, whereas machine learning, through ensemble algorithms and feature selection strategies, can effectively mine potential associations among variables (13, 14). Meanwhile, the SHAP (SHapley Additive exPlanations) interpretability framework can visualize the “black-box” model, providing a quantitative basis for clinical decision-making.

## 2 Materials and methods

### 2.1 Study design and population

This was a single-center, retrospective, cross-sectional study. From June 2024 to December 2024, patients with ED were consecutively screened at the inpatient department of Jiangsu Province Hospital of Chinese Medicine. A total of 545 patients were screened, of whom 26 were excluded for the following reasons: neurogenic ED (n = 1), history of pelvic surgery (n = 3), severe mental illness (n = 2), and recent use of hormonal or antiandrogen medications (n = 20), and 0 had two invalid monitoring nights. A total of 519 patients who completed two nights of monitoring were finally included.

Inclusion criteria were: (1) age 18–70 years; (2) having a stable partner and a chief complaint of erectile difficulty; and (3) completion of two consecutive nights of NPTR monitoring. Exclusion criteria were: neurogenic ED, history of pelvic surgery, severe mental illness, and recent use of hormonal or antiandrogen medications.

No separate mixed ED group was established in this study. Because purely psychogenic or purely organic ED is relatively uncommon in clinical practice, and because grouping in this study was based on objective NPTR outcomes, all patients meeting the inclusion criteria and completing RigiScan monitoring were classified into “effective nocturnal erection” and “no effective nocturnal erection” groups without further differentiation into psychogenic, organic, or mixed subtypes.

The study was approved by the Ethics Committee (approval number: 2024NL-309-01). Because of the retrospective design, a retrospective informed consent waiver was applied; however, all patients had signed written informed consent permitting the use of their inpatient clinical data for research. The study flowchart is shown in **Figure 1**.

**Figure 1:**
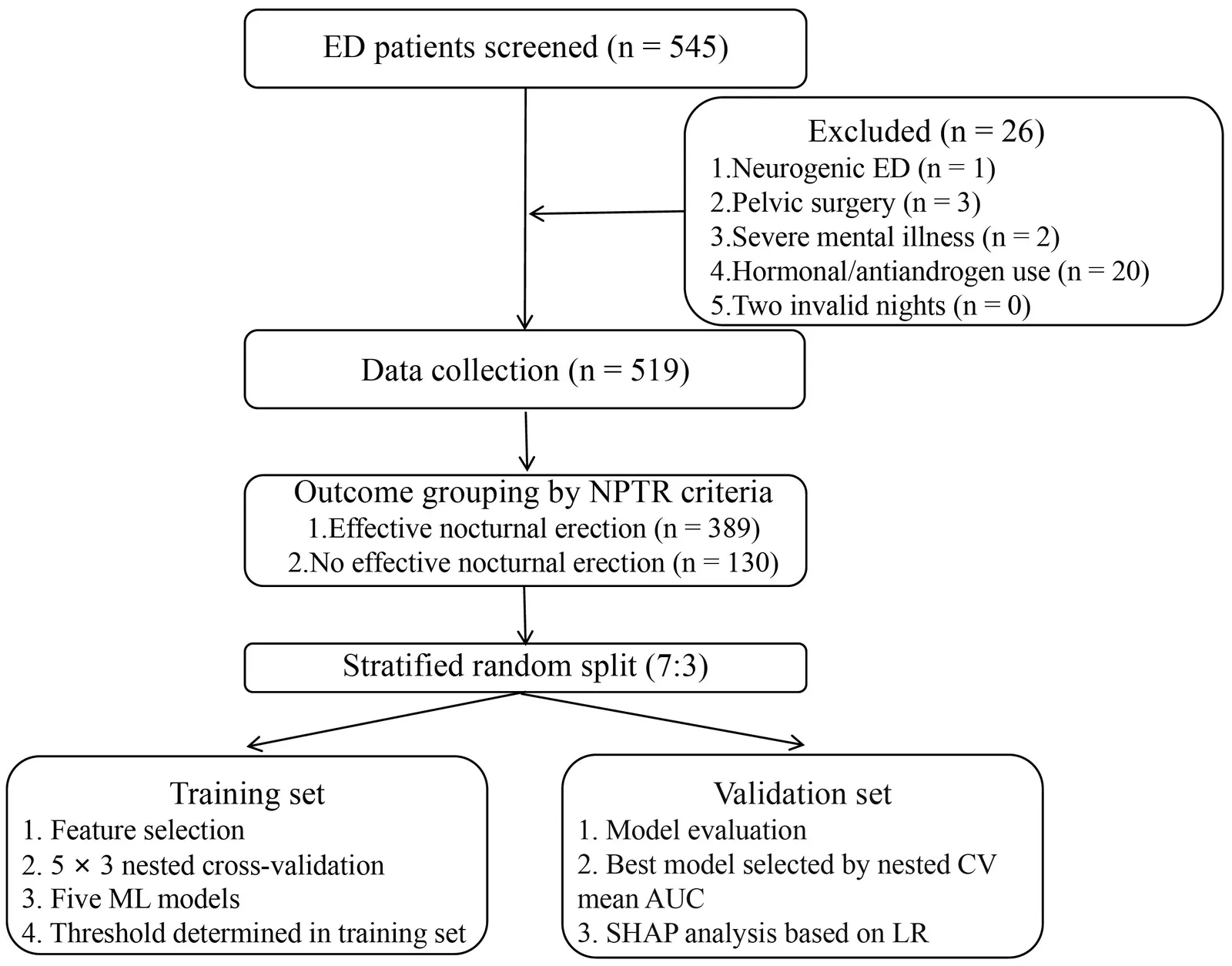
Study flowchart.

### 2.2 RigiScan grouping definition

All patients underwent two consecutive nights of monitoring using the RigiScan Plus device, and penile rigidity (%) and effective erection duration (min) were recorded. According to the standardized interpretation criteria, an effective nocturnal erection was defined as the occurrence of at least one episode with tip rigidity ≥ 60% and a duration ≥ 10 min during the two nights of monitoring; patients not meeting this criterion were classified as having no effective nocturnal erection. This binary variable was used as the outcome variable (0 = effective nocturnal erection, 1 = no effective nocturnal erection).

If only one night yielded valid data (the other night being incomplete or severely disturbed), the valid night was used for grouping, and no separate subgroup was created. Patients with two invalid nights were treated as missing data and excluded.

On the monitoring night, patients received a standardized traditional Chinese medicine foot bath (“Anshen Zuyu Fang”, composition described below) at approximately 21:00 for approximately 20 min, and were instructed to sleep at approximately 22:00. The device was fitted at approximately 22:00 and automatic recording was initiated. The following morning, between 6:00 and 7:00, patients woke up and removed the device themselves, and data were exported to the analysis software. The total recording duration per session was approximately 8 h, and the valid recording duration was 6 h.

The foot bath formula, named “Anshen Zuyu Fang” (Calming Foot Bath Formula), consisted of Cortex Cinnamomi (crushed) 6 g, Caulis Polygoni Multiflori 30 g, Flos Albiziae 12 g, Poria cum Ligno Hospite 12 g, Radix Salviae Miltiorrhizae 30 g, Radix Polygalae (honey-prepared) 6 g, Rhizoma Zingiberis 6 g, and Pericarpium Zanthoxyli 6 g. The herbs were decocted in water to an appropriate volume, and the water temperature was maintained at 38–42 °C. The foot bath was administered by uniformly trained nursing staff according to a standard protocol at approximately 21:00 on the monitoring night, with a soaking time of approximately 20 min. No other agents that might affect sleep or erection were added during the foot bath.

The raw RigiScan waveforms were reviewed by experienced technicians who were blinded to patients’ clinical data, laboratory results, and group allocation. To exclude artifacts caused by body position changes or device loosening, all raw waveforms were manually reviewed. No formal adjudication or inter-rater agreement assessment was performed; ambiguous recordings were resolved by consensus among the reviewing technicians.

### 2.3 Data collection

All clinical data were retrospectively extracted from the hospital electronic medical record system, supplemented by standardized questionnaires administered during hospitalization. The collected demographic and anthropometric variables included age, height, and weight, from which BMI was calculated. For lifestyle information, smoking and alcohol consumption were recorded as binary variables (yes/no). A history of hypertension was determined based on a prior definite diagnosis, regular use of antihypertensive medications, or a systolic blood pressure ≥ 140 mmHg and/or diastolic blood pressure ≥ 90 mmHg during hospitalization.

ED was diagnosed according to an International Index of Erectile Function-5 (IIEF-5) score ≤ 21; however, specific IIEF-5 scores were not systematically collected in this study, and the diagnosis was based on clinical complaints and IIEF-5 ≤ 21.

Psychological state assessment was prospectively completed by resident physicians within 24 hours after admission and subsequently extracted retrospectively from the electronic medical record system. The severity of anxiety and depression was assessed using the 14-item Hamilton Anxiety Rating Scale (HAMA-14) and the 24-item Hamilton Depression Rating Scale (HAMD-24), respectively. Cutoffs for HAMA-14 were defined as: < 7, no anxiety; 7–13, possible anxiety; 14–20, definite anxiety; 21–28, marked anxiety; ≥ 29, severe anxiety. Cutoffs for HAMD-24 were defined as: 0–7, no depression; 8–16, mild; 17–23, moderate; ≥ 24, severe.

Laboratory parameters were measured from peripheral venous blood samples collected on the morning after admission following an overnight fast (≥ 8 h), including fasting blood glucose (GLU), glycated hemoglobin (HbA1c), total testosterone (T), estradiol (E2), luteinizing hormone (LH), follicle-stimulating hormone (FSH), prolactin (PRL), total cholesterol (TC), triglycerides (TG), low-density lipoprotein cholesterol (LDL-C), high-density lipoprotein cholesterol (HDL-C), uric acid (UA), alanine aminotransferase (ALT), aspartate aminotransferase (AST), blood urea nitrogen (BUN), creatinine (Cr), neutrophil count (NEUT), and cystatin C (CysC). GLU was measured by the glucose oxidase method; HbA1c by high-performance liquid chromatography; T, E2, LH, FSH, PRL by chemiluminescence immunoassay. Reference intervals for each parameter are provided in the **Supplementary Material**.

Penile color Doppler duplex ultrasonography (CDDU) was performed by a sonographer with more than 5 years of experience. All patients abstained from sexual activity for approximately 1 week before examination. During the procedure, patients were placed in the supine position, and the skin was disinfected with iodophor. The corpus cavernosum below the coronal sulcus was selected as the injection site, and 15 mg papaverine was injected using a 1 mL syringe with simultaneous aspiration to avoid intravascular administration. After injection, local compression was applied, and patients were instructed to self-stimulate to achieve erection. The time to onset of erection, duration of erection, erection angle, and rigidity were recorded. After achieving erection, CDDU was performed using a L12–3 transducer, maintaining the ultrasound beam approximately parallel to the vessel and an angle of insonation of less than 60° to the direction of blood flow. The inner diameter (D), peak systolic velocity (PSV), end-diastolic velocity (EDV), and resistance index (RI) of the cavernosal arteries were measured and recorded.

After the examination, patients were informed that the injection might cause pain or a dull ache, and that a prolonged erection lasting half an hour or longer could occur. Patients were advised to drink water, walk, or engage in appropriate physical activity until detumescence. If the erection persisted for more than 1 hour or if any discomfort occurred, patients were instructed to report to the attending physician immediately. Among these, PSV ≥ 30 cm/s was considered normal, PSV < 25 cm/s suggested arterial ED, and EDV > 5 cm/s or RI < 0.8 suggested veno-occlusive dysfunction. All 519 patients completed CDDU. To avoid information leakage, Doppler parameters were not used as predictors in the machine learning models.

### 2.4 Data preprocessing and feature selection

The dataset was divided into a training set (n = 364) and an internal holdout validation set (n = 155) using stratified random sampling at a 7:3 ratio. All feature selection was performed exclusively within the training set to prevent information leakage.

Data were manually collected and entered by researchers from the electronic medical record system. All candidate variables had no missing values (0% missing), so no imputation was performed. No standardization of continuous variables was applied in this study because the machine learning models used (LR, RF, LightGBM, XGBoost) are not sensitive to variable scaling. A global random seed of 88888 was set for all models.

Three methods were employed, and their intersection was taken: (1) univariate logistic regression (P < 0.05); (2) the Boruta algorithm (based on random forest, 100 iterations, retaining “confirmed” variables); and (3) least absolute shrinkage and selection operator (LASSO) regression (10-fold cross-validation to determine the minimum λ, retaining variables with non-zero coefficients). The final intersection was used as the input features for subsequent modeling. To avoid information leakage, RigiScan-related parameters (e.g., TET, NEE, rigidity, Doppler parameters) were excluded from candidate predictors.

### 2.5 Machine learning model construction and evaluation

Five binary classification models were constructed based on the training set. All models used fixed default parameters: (1) LR: binomial generalized linear model with a logit link, no regularization; (2) RF: ntree = 1000, mtry = square root of the number of features; (3) SVM: radial basis function kernel, C = 1, gamma = 1/feature dimension, probability estimation enabled; (4) LightGBM: histogram-based algorithm, objective = binary log loss, num_leaves = 31, learning_rate = 0.1, rounds = 200; (5) XGBoost: objective = binary:logistic, max_depth = 6, eta = 0.1, rounds = 200. The classification threshold was determined in the training set using the Youden index and fixed in the validation set. The positive class in this study was “no effective nocturnal erection” (coded as 1). Model performance was assessed using area under the receiver operating characteristic curve (AUC), accuracy, sensitivity, specificity, precision, F1 score, and geometric mean (G-mean). The 95% confidence interval (CI) for the AUC was estimated using bootstrap resampling of the validation set predictions (1,000 resamples); feature selection and model training were not repeated within each bootstrap sample. In addition, 5 × 3 nested cross-validation was performed, with feature selection and model training repeated inside each training fold to evaluate the robustness of the entire model development process. The software environment was R 4.5.1. Major package versions included tidyverse 2.0.0, glmnet 4.1-10, randomForest 4.7-1.2, pROC, caret, and fastshap (15).

### 2.6 Model interpretability (SHAP)

The model with the highest mean AUC in nested cross-validation (LR) was selected as the best model, and the fastshap package was used to calculate its SHAP values. The SHAP prediction function was predict(model, newdata, type = “response”), with the positive class defined as “no effective nocturnal erection.” Feature importance bar charts, beeswarm plots, and dependence plots for continuous variables were generated to quantify the contribution direction, relative importance, and potential threshold effects of each feature on the prediction. In addition, SHAP stability was assessed using five resampling runs with different random seeds.

### 2.7 Statistical analysis

For baseline characteristics, continuous variables that did not follow a normal distribution (Shapiro-Wilk test) were expressed as median (interquartile range) and compared between groups using the Mann-Whitney U test. Categorical variables were expressed as number (percentage) and compared using the chi-square test. All statistical analyses and machine learning modeling were performed using R version 4.5.1 and SPSS 26.0. A two-sided P < 0.05 was considered statistically significant.

## 3 Results

### 3.1 Baseline characteristics

A total of 519 patients were enrolled, with 389 in the effective erection group and 130 in the non-effective erection group. Baseline comparison (**Table 1**) showed that patients in the non-effective erection group were older, had a higher BMI, and exhibited higher proportions of hypertension and smoking. Regarding laboratory parameters, the non-effective erection group had significantly higher levels of fasting blood glucose (GLU), HbA1c, neutrophil count, and FSH compared with the effective group. Although the HAMA score differed between the two groups, the median scores in both groups fell within the mild anxiety range, which limited the clinical significance of this difference. No significant differences were observed between the two groups in alcohol consumption history, blood lipid profiles, uric acid, hepatic and renal function, total testosterone, estradiol, or prolactin (all P > 0.05). For Rigiscan parameters, the non-effective erection group exhibited significantly fewer erectile events, lower tip and base rigidity, reduced tumescence, and shorter effective duration than the effective group. In addition, penile color Doppler ultrasound parameters (PSV, peak systolic velocity; EDV, end-diastolic velocity; RI, resistance index) indicated poorer arterial supply function in the non-effective erection group (P < 0.05).

**Table 1:** Baseline characteristics of the study participants.

Variable	Effective erection group (n=389)	Ineffective erection group (n=130)	P value
Age (years)	34.00 (29.00, 42.00)	36.00 (31.00, 46.25)	0.001
ALT (U/L)	22.00 (14.00, 32.00)	21.00 (14.00, 29.00)	0.383
AST (U/L)	20.00 (17.00, 25.00)	20.00 (17.00, 25.00)	0.617
IGF-1 (ng/mL)	169.00 (137.50, 201.50)	164.00 (131.25, 196.25)	0.234
BMI (kg/m²)	24.80 (22.60, 27.17)	26.43 ± 3.58	<0.001
UA (μmol/L)	374.00 (324.50, 419.50)	359.00 (311.75, 417.75)	0.292
LDL (mmol/L)	2.90 ± 0.71	2.89 ± 0.85	0.550
HDL (mmol/L)	1.07 (0.91, 1.26)	1.05 (0.92, 1.20)	0.365
TG (mmol/L)	1.40 (0.94, 2.01)	1.40 (0.94, 2.01)	0.388
TC (mmol/L)	4.47 ± 0.84	4.49 ± 0.97	0.870
T (ng/dL)	443.52 (329.76, 560.56)	413.44 (307.44, 542.16)	0.115
E2 (ng/dL)	30.00 (24.00, 36.00)	30.82 ± 10.37	0.481
LH (mIU/mL)	5.40 (4.17, 6.93)	6.03 (4.31, 8.07)	0.055
FSH (mIU/mL)	4.16 (2.98, 5.80)	5.00 (3.46, 6.67)	0.001
PRL (ng/dL)	18.71 (14.92, 23.52)	17.46 (14.18, 24.72)	0.521
SBP (mmHg)	127.00 (118.50, 133.50)	125.50 (119.00, 136.00)	0.703
DBP (mmHg)	81.00 (76.00, 87.00)	83.00 (77.00, 88.00)	0.117
GLU (mmol/L)	4.70 (4.45, 5.05)	4.92 (4.60, 5.71)	<0.001
HbA1c (%)	5.50 (5.30, 5.70)	5.60 (5.40, 6.23)	<0.001
Hb (g/L)	152.55 ± 10.09	154.45 ± 11.07	0.069
PLT (×10^9^/L)	219.00 (190.00, 255.00)	216.00 (181.75, 257.75)	0.536
NEUT (×10^9^/L)	3.20 (2.50, 3.80)	3.30 (2.70, 3.90)	0.017
ALB (g/L)	44.00 (43.00, 46.00)	44.00 (43.00, 45.00)	0.077
BUN (mmol/L)	5.10 (4.30, 5.80)	5.10 (4.40, 5.90)	0.459
Cr (μmol/L)	75.10 ± 10.29	74.42 ± 12.49	0.573
CysC (mg/L)	0.94 (0.87, 1.01)	0.95 (0.87, 1.04)	0.214
HAMD (score)	12.00 (7.00, 17.00)	9.50 (5.00, 18.00)	0.241
HAMA (score)	12.00 (8.00, 18.00)	11.00 (7.00, 17.00)	0.027
TET (minutes)	70.75 (49.94, 101.75)	10.25 (2.50, 26.25)	<0.001
NEE (count)	2.00 (1.00, 4.00)	0.00 (0.00, 0.00)	<0.001
MGEH (%)	63.00 (55.00, 71.00)	41.00 (1.00, 53.00)	<0.001
MGTR (%)	37.97 ± 9.52	25.00 (3.00, 33.00)	<0.001
T_Glans_H60 (min)	44.25 (26.50, 69.63)	3.00 (0.00, 5.50)	<0.001
RAU_G (U)	46.00 (30.00, 66.00)	5.00 (0.00, 10.00)	<0.001
TAU_G (U)	27.00 (18.00, 42.00)	3.00 (1.00, 7.00)	<0.001
MBEH (%)	64.00 (55.00, 72.00)	47.00 (6.00, 60.00)	<0.001
MBTR (%)	36.00 (31.00, 41.00)	26.00 (20.00, 32.00)	<0.001
T_Base_H60 (min)	44.75 (25.75, 70.00)	3.50 (0.00, 8.50)	<0.001
RAU_B (U)	47.50 (31.00, 68.00)	6.00 (1.00, 13.00)	<0.001
TAU_B (U)	28.00 (17.00, 39.00)	3.00 (1.00, 8.00)	<0.001
L-PSV (cm/s)	27.15 (17.38, 37.30)	19.70 (14.70, 30.90)	<0.001
L-EDV (cm/s)	0.40 (0.35, 0.92)	0.43 (0.38, 3.01)	0.011
L-RI	0.98 (0.94, 0.98)	0.97 (0.82, 0.98)	<0.001
R-PSV (cm/s)	27.55 (18.20, 35.78)	23.30 (15.10, 31.50)	<0.001
R-EDV (cm/s)	0.43 (0.38, 2.45)	0.47 (0.39, 2.81)	0.034
R-RI	0.98 (0.89, 0.98)	0.97 (0.83, 0.98)	0.001
Smoking			<0.001
0	317 (81.49%)	79 (60.77%)	
1	72 (18.51%)	51 (38.23%)	
Alcohol consumption			0.147
0	272 (69.92%)	82 (63.08%)	
1	117 (30.08%)	48 (36.92%)	
Hypertension history			<0.001
0	335 (86.12%)	84 (64.62%)	
1	54 (13.88%)	46 (35.38%)	

Continuous variables are presented as median (IQR) or mean ± SD, and categorical variables as n (%). P values were calculated using the Mann-Whitney U test for continuous variables and the chi-square test for categorical variables. RigiScan parameters are presented in **Table 1** only to describe group characteristics and were not used as predictors in the machine learning models.

### 3.2 Feature selection results

In the training set, univariate logistic regression initially identified 11 candidate variables (HbA1c, GLU, Smoke, Hypertension, Age, FSH, BMI, PRL, Drunk, HAMA, NEUT). The Boruta algorithm confirmed six variables (HbA1c, GLU, Smoke, Hypertension, Age, BMI; **Figure 2**). LASSO regression retained 13 variables with non-zero coefficients (**Figures 3**, **4**). The intersection of the three methods ultimately selected six predictors: HbA1c, GLU, Smoke, Hypertension, Age, and BMI.

**Figure 2:**
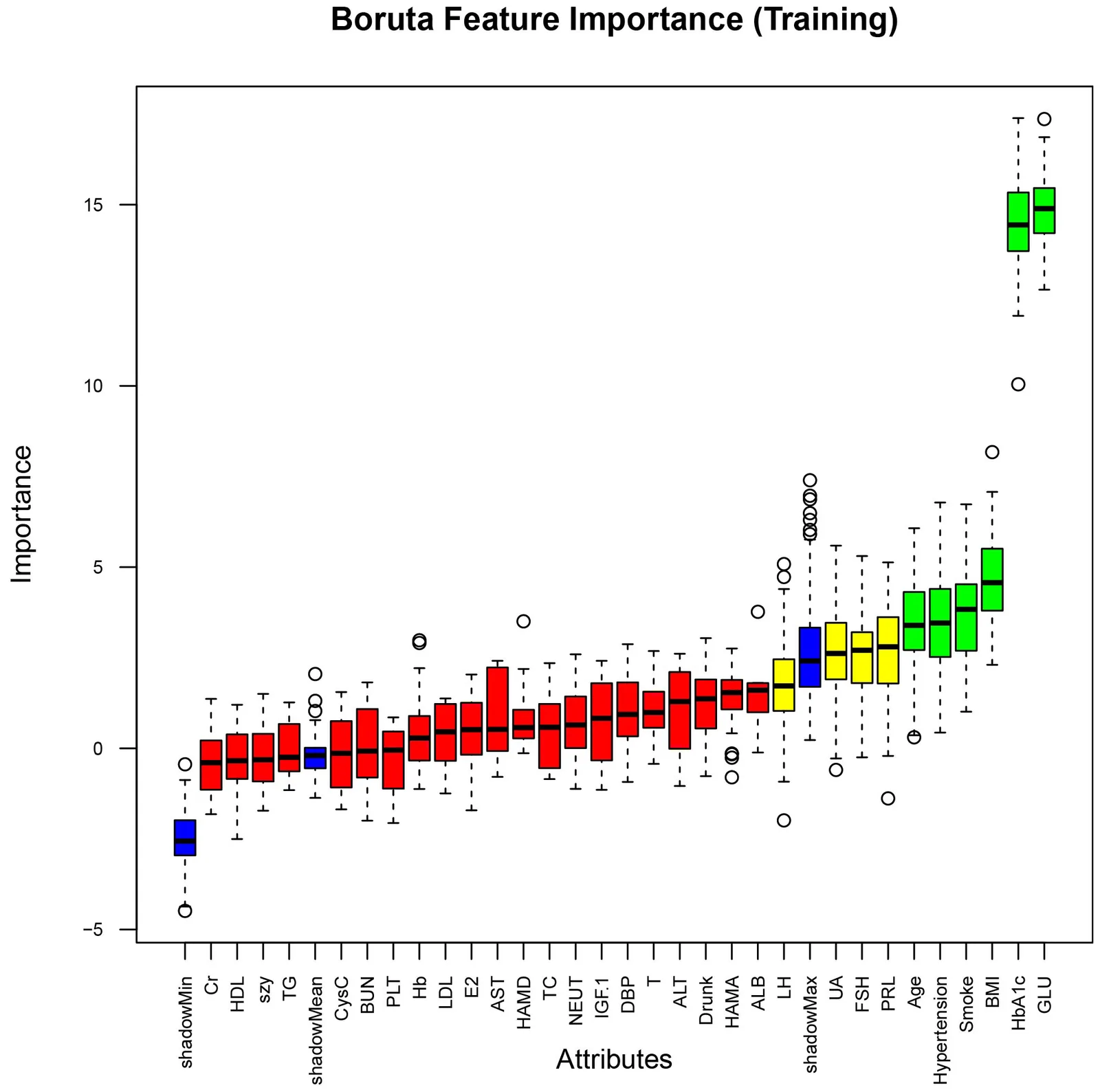
Feature selection using the Boruta algorithm in the training set. Green box plots represent features confirmed as important; red box plots represent rejected features; blue box plots represent shadow features; yellow box plots represent tentative features. Six variables were confirmed: HbA1c, GLU, Smoke, Hypertension, Age, and BMI.

**Figure 3:**
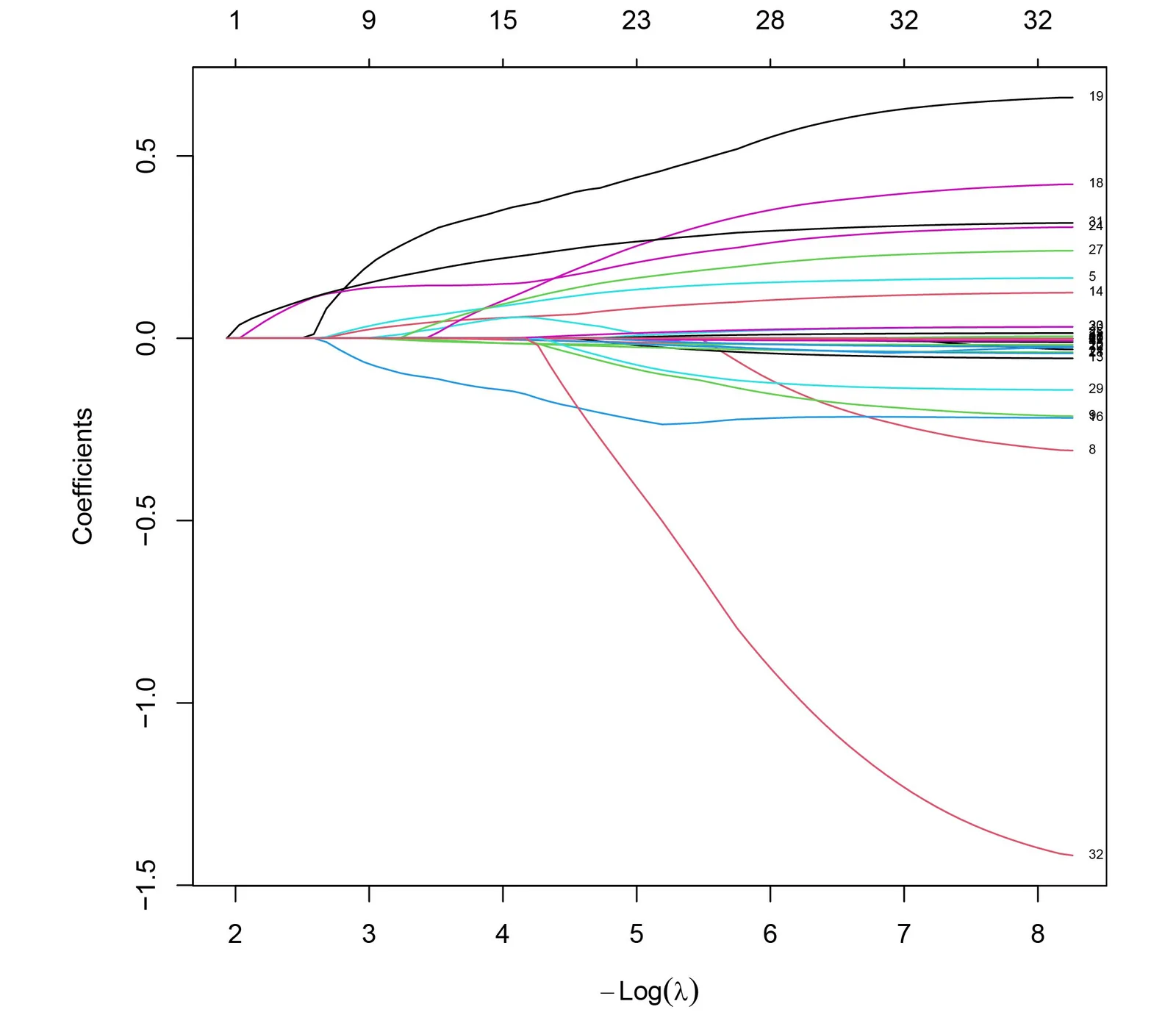
LASSO coefficient shrinkage path plot in the training set. The horizontal axis represents log(λ), and the vertical axis represents the regression coefficients. Each colored curve corresponds to a clinical indicator. As λ increases, coefficients gradually shrink toward zero. At the λ.min value, 13 variables with non-zero coefficients were retained.

**Figure 4:**
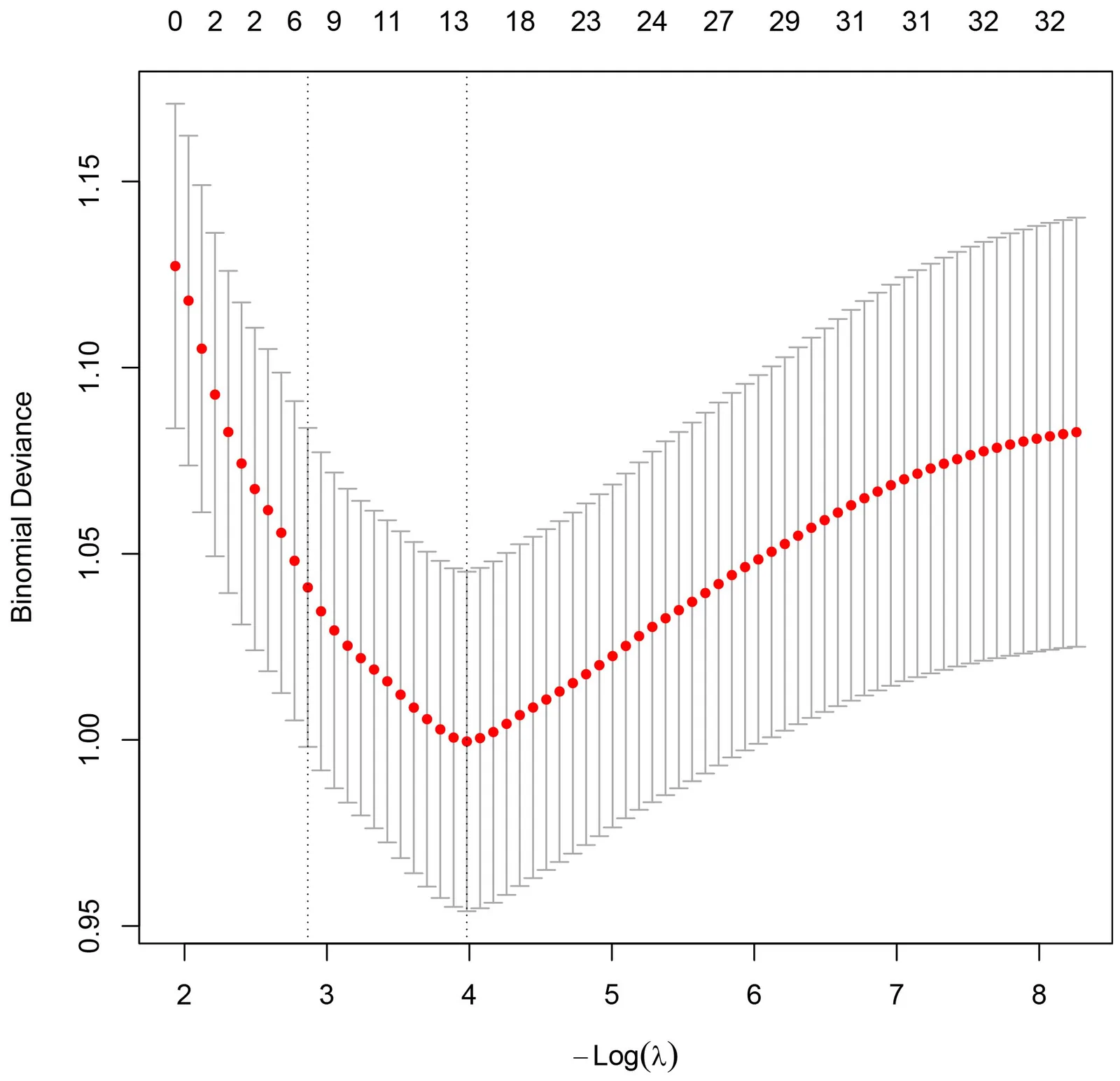
LASSO regression cross-validation error curve. The horizontal axis represents log(λ), and the vertical axis represents binomial deviance. The two dashed vertical lines correspond to λ.min and λ.1se. In this study, λ.min was selected, at which point 13 variables with non-zero coefficients were retained.

### 3.3 Model performance comparison

The discriminative performance of all machine learning models in the internal validation set is summarized in **Table 2**. In the internal validation set, LR achieved the highest observed AUC (0.779, 95% CI 0.692–0.861), followed by LGBM (0.718, 95% CI 0.621–0.815), XGB (0.700, 95% CI 0.606–0.791), RF (0.699, 95% CI 0.603–0.794), and SVC (0.646, 95% CI 0.528–0.760; **Figure 5**). LR showed the lowest Brier score (0.151) and calibration slope (1.258) and intercept (0.308) closest to ideal values (**Figure 6**). DeLong’s test showed that LR significantly outperformed RF (p = 0.028) and SVC (p = 0.009), and was not significantly different from LGBM (p = 0.160) or XGB (p = 0.072). In decision curve analysis, LR provided the highest net benefit across clinically relevant thresholds (**Figure 7**). In 5 × 3 nested cross-validation, LR also had a higher mean AUC than RF (0.726 ± 0.052 vs 0.666 ± 0.034).

**Table 2:** Discriminative performance of each machine learning model in the internal validation set.

Model	AUC	Accuracy	Sensitivity	Specificity	PPV	NPV	F1	G-mean	Brier	Brier-rms	Threshold	Cal-intercept	Cal-slope
LR	0.779	0.729	0.59	0.776	0.469	0.849	0.523	0.676	0.151	0.151	0.275	0.308	1.258
RF	0.699	0.748	0.385	0.871	0.5	0.808	0.435	0.579	0.169	0.169	0.374	-0.285	0.580
SVC	0.646	0.697	0.59	0.733	0.426	0.842	0.495	0.657	0.166	0.166	0.205	1.391	2.044
LGBM	0.718	0.768	0.462	0.871	0.545	0.828	0.5	0.634	0.178	0.178	0.475	-0.469	0.286
XGB	0.700	0.735	0.385	0.853	0.469	0.805	0.423	0.573	0.188	0.188	0.384	-0.463	0.341

LR, logistic regression; LGBM, light gradient boosting machine; RF, random forest; SVC, support vector machine; XGB, extreme gradient boosting machine; AUC, area under the receiver operating characteristic curve; PPV, positive predictive value; NPV, negative predictive value; F1, F1 score; G-mean, geometric mean; Brier, Brier score; Cal_Intercept, calibration intercept; Cal_Slope, calibration slope; Threshold, classification threshold. The positive class was defined as 1 = no effective nocturnal erection, and the negative class as 0 = effective nocturnal erection. For all models, the classification threshold was determined in the training set using the Youden index and then fixed in the validation set. A lower Brier score indicates better predictive accuracy; a calibration slope closer to 1 and a calibration intercept closer to 0 indicate better calibration. Calibration slope and intercept were calculated based on predicted probabilities in the validation set.

**Figure 5:**
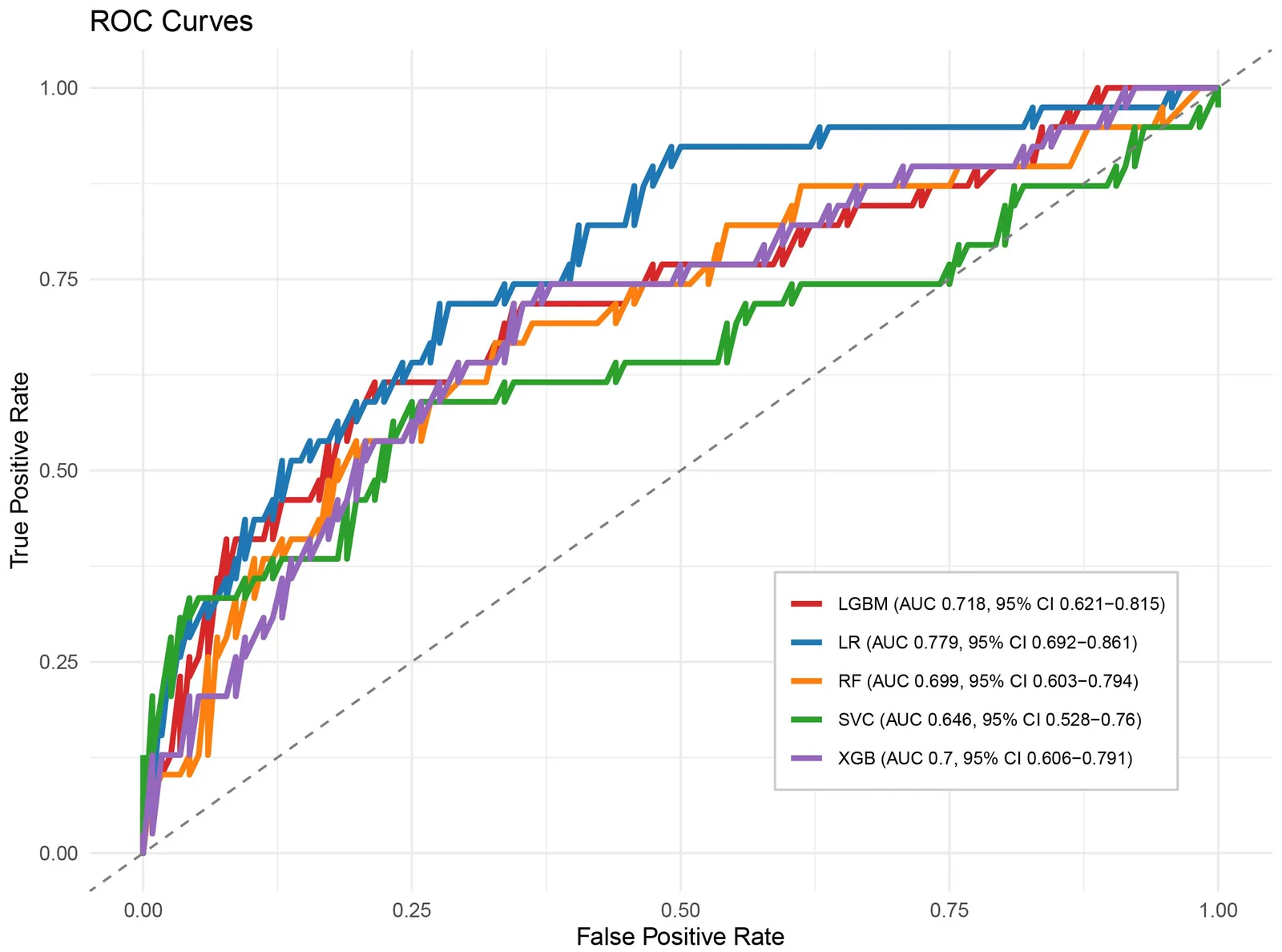
ROC curves of the five machine learning models in the internal validation set. LR showed the highest AUC (0.779, 95% CI 0.692–0.861), followed by LGBM (0.718, 95% CI 0.621–0.815), XGB (0.700, 95% CI 0.606–0.791), RF (0.699, 95% CI 0.603–0.794), and SVC (0.646, 95% CI 0.528–0.760). The diagonal line represents random prediction.

**Figure 6:**
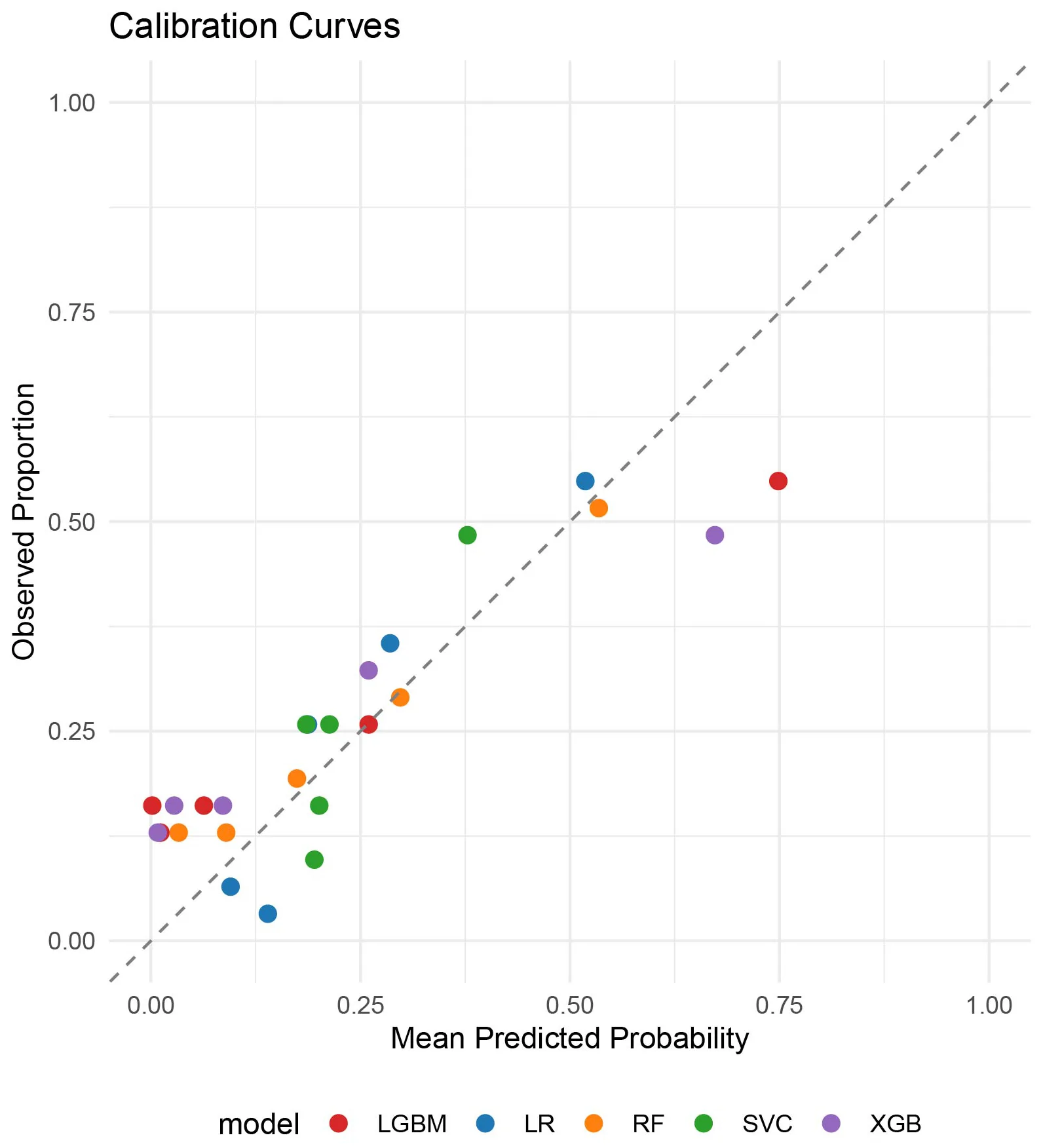
Calibration curves of all models in the internal validation set. Points represent deciles of predicted probability; the dashed line represents perfect calibration. LR showed the best calibration, with a Brier score of 0.151 and a calibration slope of 1.258.

**Figure 7:**
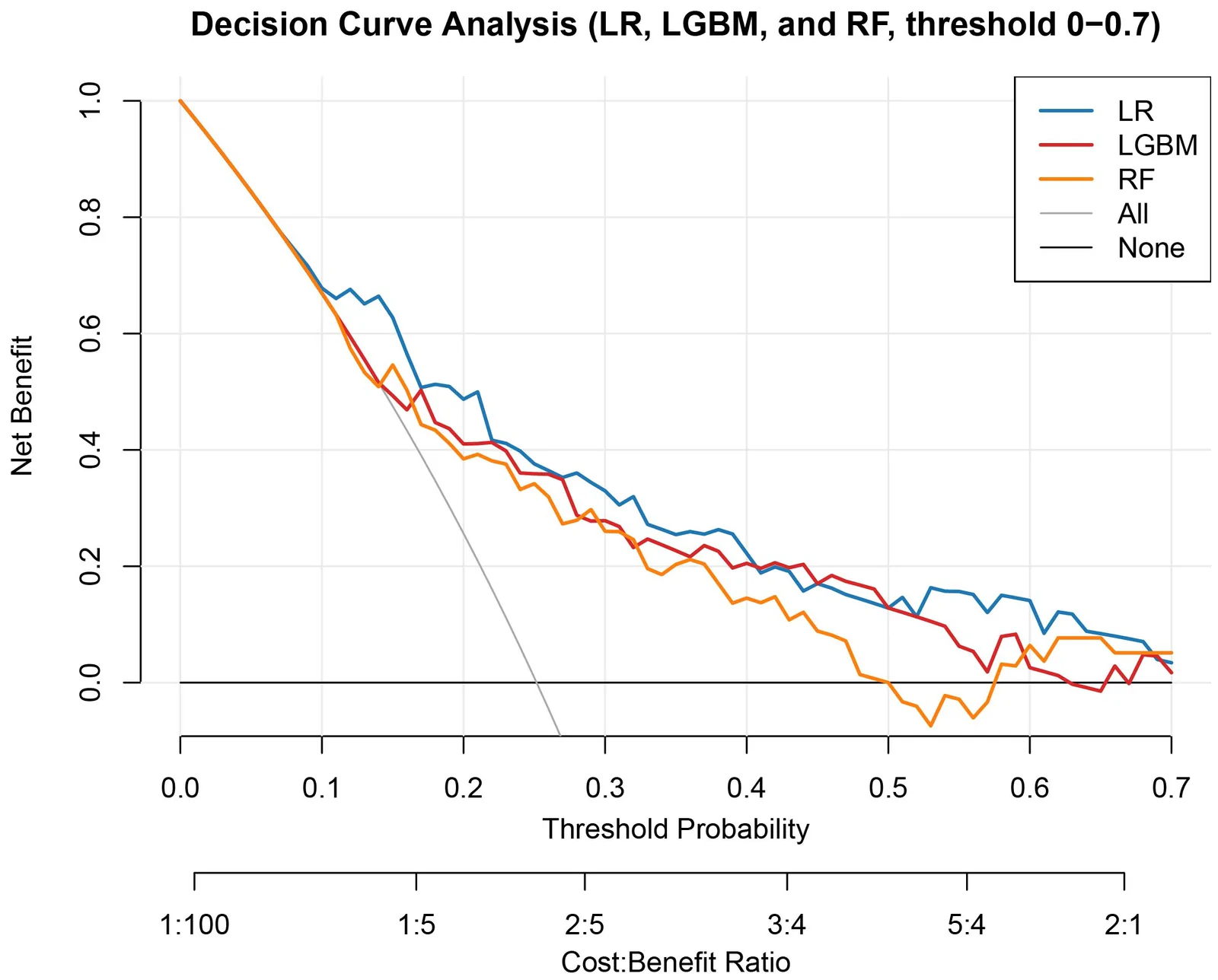
Decision curve analysis of LR, LGBM, and RF in the internal validation set. The horizontal axis represents threshold probability, and the vertical axis represents net benefit. LR showed the highest net benefit across clinically relevant threshold probabilities.

The classification threshold in the validation set was 0.275 for LR, 0.374 for RF, and 0.475 for LightGBM. The PPV and NPV were 0.469 and 0.849 for LR, 0.500 and 0.808 for RF, and 0.545 and 0.828 for LightGBM, respectively. The 95% CIs for PPV and NPV were not calculated due to the limited number of positive events in the validation set.

### 3.4 SHAP model interpretation

SHAP analysis based on the LR model identified BMI, GLU, and Age as the top three contributors, followed by Hypertension, HbA1c, and Smoking (**Figure 8**). The beeswarm plot confirmed that higher BMI, GLU, and HbA1c values were associated with positive SHAP values (**Figure 9**). Dependence plots revealed approximately monotonic positive associations between BMI, GLU, and HbA1c and the predicted probability of no effective nocturnal erection (**Figure 10**). No clear threshold effect was observed in the LR model.

**Figure 8:**
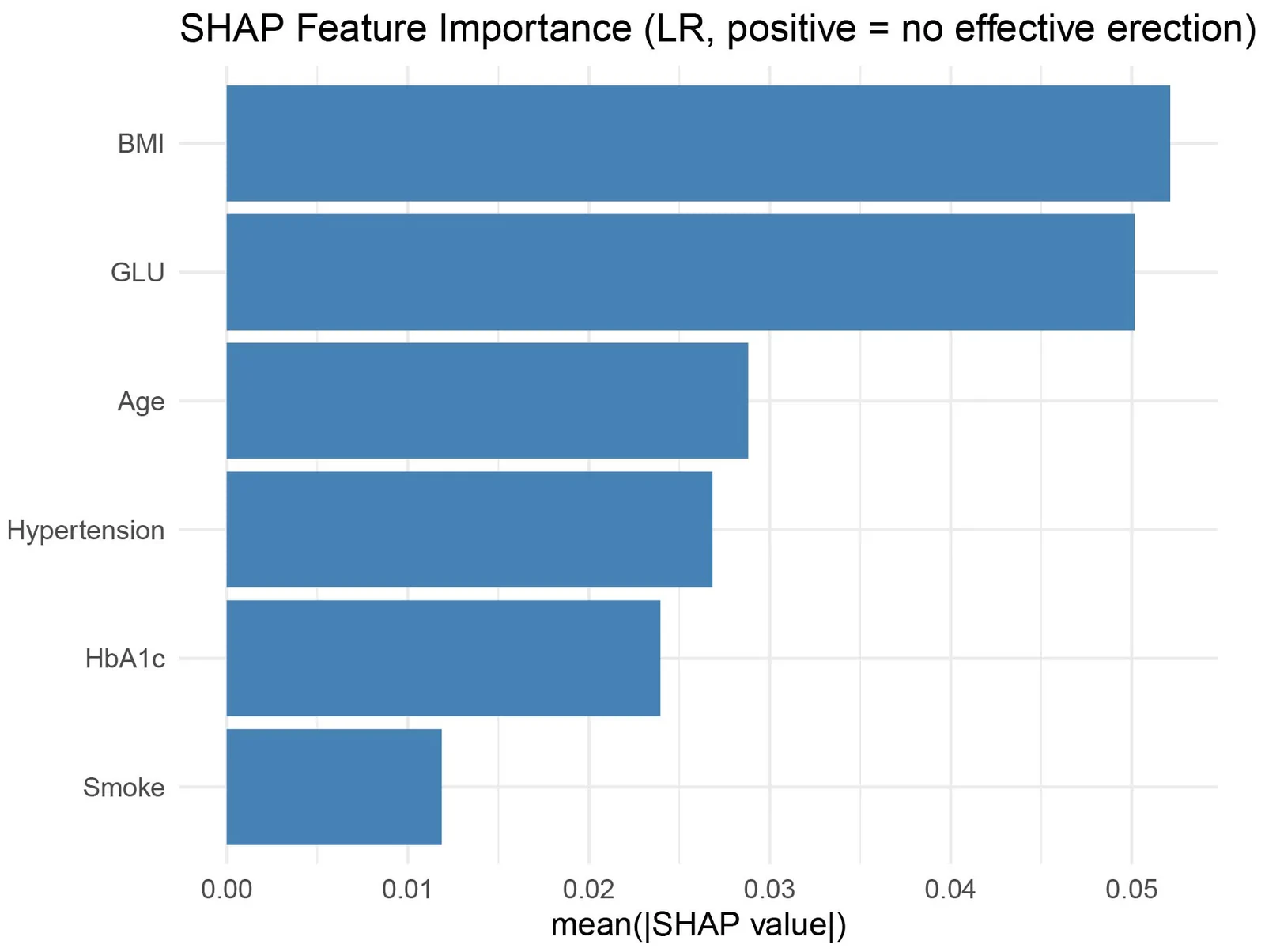
Global feature importance based on the LR model using SHAP. The horizontal axis represents the mean absolute SHAP value of each feature. BMI contributed the most, followed by GLU, Age, Hypertension, HbA1c, and Smoking.

**Figure 9:**
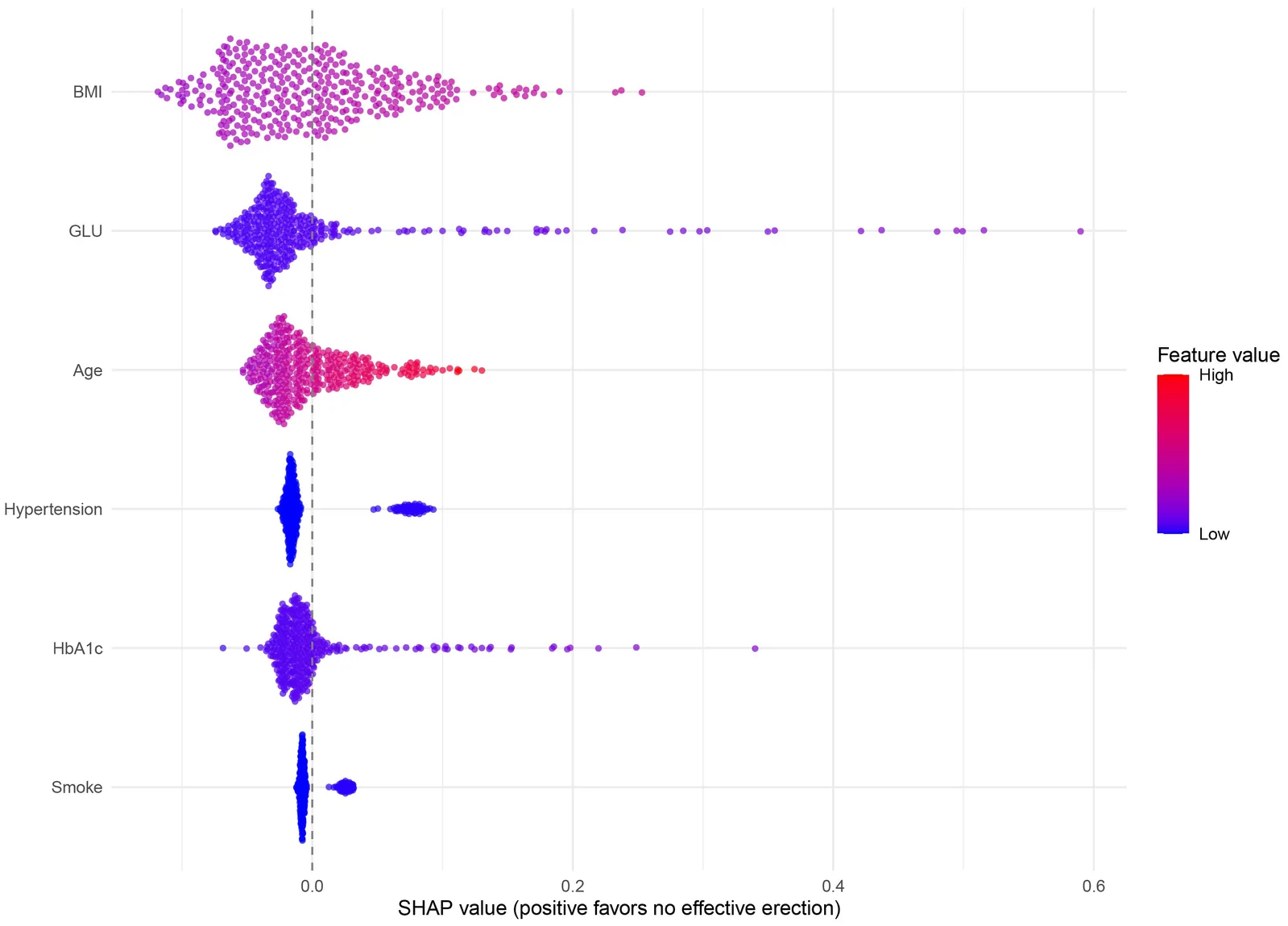
SHAP beeswarm plot of feature impacts based on the LR model. Each point represents a sample. Positive SHAP values indicate that the feature pushes the model’s prediction toward “no effective nocturnal erection”; negative values indicate the opposite direction. The color gradient from blue to red reflects feature values from low to high.

**Figure 10:**
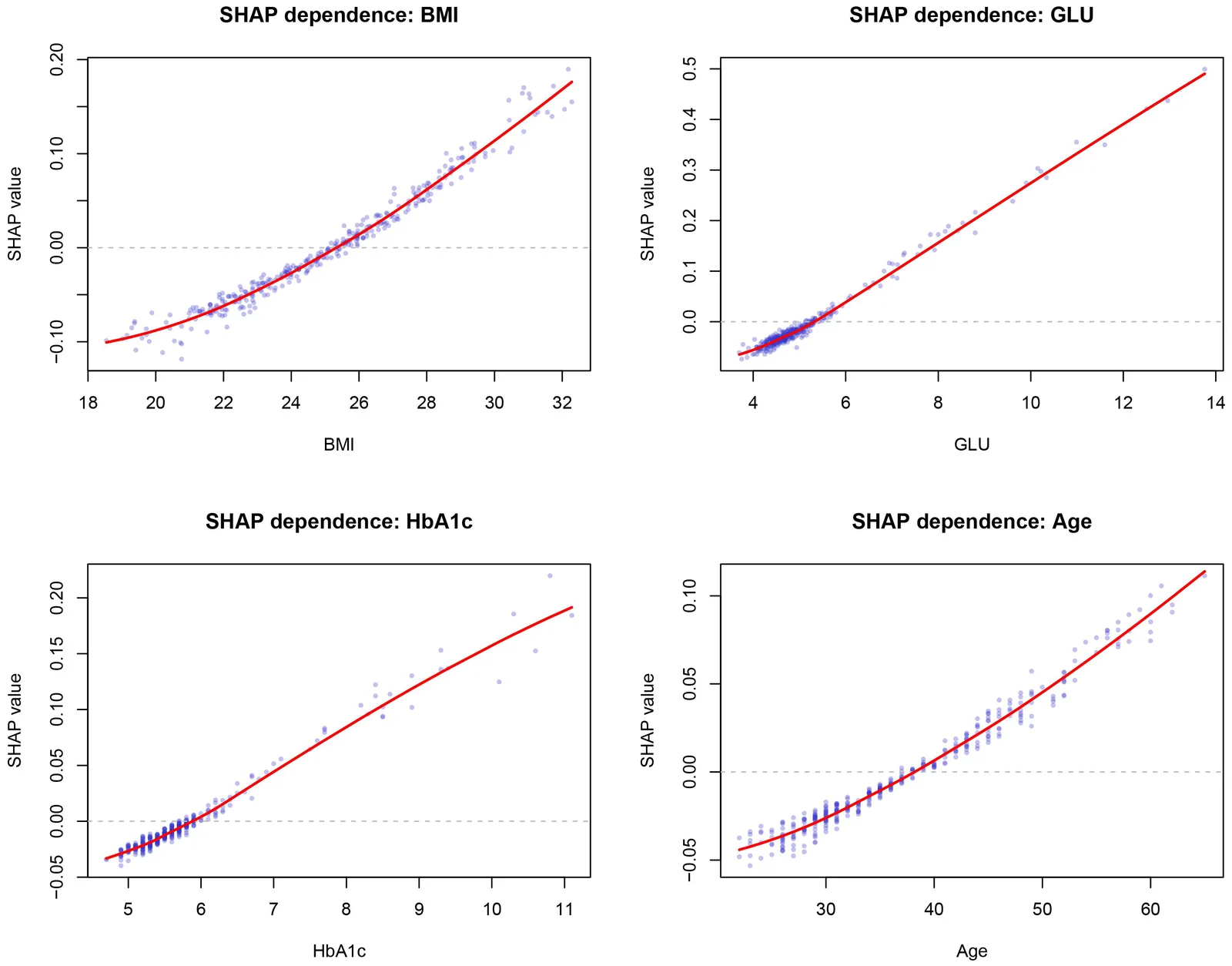
SHAP dependence plots for BMI, GLU, HbA1c, and Age based on the LR model. The horizontal axis shows the measured value of each indicator, and the vertical axis shows the corresponding SHAP contribution value. Blue points represent individual samples, and the solid red line represents the LOESS-smoothed trend. Approximate monotonic positive associations were observed for BMI, GLU, and HbA1c.

## 4 Discussion

This study, based on objective RigiScan-based phenotypic classification combined with machine learning feature selection and multi-model comparison, identified six clinical indicators associated with failure to meet the NPTR efficacy criterion: HbA1c, GLU, Smoke, Hypertension, Age, and BMI. In the internal holdout validation set, the LR model achieved the highest observed AUC (0.779, 95% CI 0.692–0.861), followed by LGBM (0.718, 95% CI 0.621–0.815), XGB (0.700, 95% CI 0.606–0.791), RF (0.699, 95% CI 0.603–0.794), and SVM (0.646, 95% CI 0.528–0.760). In 5 × 3 nested cross-validation, LR also had a higher mean AUC than RF (0.726 ± 0.052 vs 0.666 ± 0.034). DeLong’s test showed that LR significantly outperformed RF (p = 0.028) and SVM (p = 0.009), and was not significantly different from LGBM (p = 0.160) or XGB (p = 0.072). LR also showed the lowest Brier score (0.151) and a calibration slope closest to 1 (1.258), indicating better calibration than the other models. These findings suggest that linear models may be more robust than ensemble algorithms in low-dimensional, small-sample settings, and that metabolic indicators may have an approximately linear association with failure to meet NPTR criteria.

Multiple algorithms consistently identified glucose metabolism indicators, highlighting a close association between hyperglycemia and failure to meet NPTR criteria. Erectile function depends on the coordinated effects of neurovascular and psychological factors, with the final common pathway being the contraction-relaxation balance of cavernous smooth muscle (5, 16). Vascular disease is a major potential etiology of organic erectile impairment (17). A core pathological component of metabolic syndrome-associated ED is penile vascular endothelial dysfunction; hyperglycemia, obesity, hypertension, and dyslipidemia have been implicated in synergistic impairment of cavernous vascular endothelium, involving pathways such as nitric oxide/cyclic guanosine monophosphate (NO/cGMP), Ras homolog family member A/Rho-associated coiled-coil containing protein kinase (RhoA/ROCK), endothelial-derived vasoactive factors, testosterone levels, oxidative stress, and programmed cell death (18). Persistent hyperglycemia has been associated with impaired vasodilatory signaling, excessive smooth muscle contraction, and veno-occlusive dysfunction (19). At the molecular level, hyperglycemia has been linked to increased formation of advanced glycation end products, suppressed endothelial nitric oxide synthase (eNOS) phosphorylation, eNOS uncoupling, reduced NO bioavailability, activation of RhoA/ROCK, enhanced vasoconstrictive effects of endothelin-1 and angiotensin II, increased release of inflammatory cytokines such as tumor necrosis factor-alpha (TNF-α), and pro-apoptotic signaling, which may ultimately impair cavernous smooth muscle relaxation and blood perfusion (18, 20–23). Therefore, abnormal glucose metabolism is not only a manifestation of metabolic syndrome but is also associated with the presence and severity of organic ED.

SHAP dependence plots based on the LR model showed approximately monotonic positive associations between BMI, GLU, and HbA1c and the predicted probability of no effective nocturnal erection. No clear threshold effect was observed in the LR model; the previously reported inflection point of HbA1c around 6.0% in tree-based models may reflect model-specific behavior rather than a definite biological threshold. This finding suggests that elevated HbA1c, even below the diagnostic threshold for diabetes, may be associated with microvascular and endothelial dysfunction in erectile impairment. It supports the routine inclusion of HbA1c testing in the etiological evaluation of ED, as well as its use as a target for glycemic management and metabolic stratification, but the precise threshold should be interpreted with caution.

BMI was another important feature. In the LR-based SHAP analysis, BMI contributed the most to the model output. SHAP showed that BMI was generally positively associated with the absence of effective erection, suggesting that insulin resistance related to visceral fat, chronic inflammation, endothelial dysfunction, and increased conversion of testosterone to estradiol in overweight/obese individuals may be associated with failure to meet NPTR criteria (24–28). Mechanistically, obesity has been implicated in further impairment of endothelium-dependent vasodilation and NO/cGMP signaling through adipokine dysregulation, chronic low-grade inflammation, oxidative stress, and an imbalance in the testosterone/estrogen ratio (18). Previous studies also support a positive association between obesity and ED. For example, a cross-sectional study of 878 male patients attending urology clinics in central China reported an odds ratio of 1.78 for ED in the obese group (95% CI: 1.10–2.90), with a stronger association for moderate-to-severe ED (OR = 2.51, 95% CI: 1.24–5.09) (29). The present study used objective RigiScan-based grouping and provided new data supporting this association at the level of NPTR outcomes, suggesting that BMI should be considered an important indicator for metabolic risk monitoring. Therefore, obesity-related ED may not be driven solely by body weight itself but may be more closely related to metabolic-vascular networks involving insulin resistance, low-grade inflammation, low testosterone, and endothelial dysfunction.

Compared with the previous discriminant model based on intracavernosal injection (ICI) plus CDDU (30), the relevant factors retained in this study showed a distinct phenotypic difference. The previous ICI+CDDU model retained age, hypertension, and testosterone as the main predictors, suggesting that systemic vascular aging, atherosclerosis, and androgen deficiency are closely associated with pharmacologically induced cavernosal hemodynamic impairment. In contrast, SHAP analysis in the present study identified BMI, GLU, and Age as the top three contributors, followed by Hypertension, HbA1c, and Smoking. Notably, testosterone did not enter the final six-variable model in this study, whereas the contributions of BMI and glycemic indicators (GLU and HbA1c) were substantially enhanced. This difference may be attributed to the distinct pathophysiological dimensions captured by the two examination methods: ICI+CDDU assesses peak systolic velocity and veno-occlusive function after pharmacologically induced vascular stimulation, primarily reflecting vascular structural reserve and age-related arteriosclerosis; whereas RigiScan records nocturnal erectile hardness and duration during natural sleep, integrating central neural drive, sleep architecture integrity, and long-term metabolic adaptation of cavernous smooth muscle. Consequently, metabolic burden, insulin resistance, and chronic hyperglycemia play a more prominent role in its prediction. Furthermore, although Smoke and Hypertension were included in the final model, their SHAP rankings were relatively low, suggesting that the impact of traditional vascular risk factors on NPTR failure may be partially masked by metabolic factors. In the previous ICI model, age was an immutable intrinsic attribute; in contrast, BMI, GLU, and HbA1c in the present model are modifiable metabolic targets, providing a potential direction for applying prediction results to glycemic control and weight management.

In this study, HAMA (anxiety score) did not enter the feature set of the Rigiscan-based model, whereas this variable was retained in the previous CDDU model. This difference may suggest inherent differences in the sensitivity of the two examination methods to psychological factors. Although ICI has a direct drug-induced vasodilatory effect, situational anxiety at the visit and psychological reactions to expected examination results may still be associated with bias in CDDU measurements, possibly involving sympathetic nervous system activation (31). In contrast, Rigiscan is performed during nighttime sleep in a home-like environment, and nocturnal erections are mainly driven by parasympathetic output and central nervous system regulation, which may make it less susceptible to anxiety-mediated sympathetic fluctuations than awake pharmacologically stimulated testing (32). Therefore, the retention of HAMA in the CDDU model and its exclusion from the RigiScan model may be related to the different neurophysiological bases of the two examinations, rather than a substantial difference in the influence of psychological factors on NPTR outcomes. This phenomenon also indirectly supports the methodological advantage of RigiScan in reducing psychological confounding factors.

The gut-brain axis (GBA) is a bidirectional communication network between the gut and the central nervous system. Gut microbiota can influence brain function through short-chain fatty acids, hormones, neurotransmitters, and immune signals. Tryptophan (Trp) is the sole precursor of 5-hydroxytryptamine (5-HT) and can cross the blood-brain barrier, where it is converted to 5-HT by tryptophan hydroxylase 2 (Tph2); approximately 90% of 5-HT is synthesized in the gut, and short-chain fatty acids can enhance Trp hydroxylase activity in enterochromaffin cells, whereas gut microbiota dysbiosis may reduce Trp/5-HT synthesis and subsequently affect mood, sleep, ejaculation, and erectile regulation (33). Tph2 gene polymorphisms have been associated with premature ejaculation risk, and 5-HT receptor subtypes are also involved in ejaculation regulation. Although this study focused on failure to meet NPTR criteria and RigiScan nocturnal erectile monitoring, the GBA may still influence NPTR through central neural drive, sleep architecture, anxiety and depression states, and autonomic nervous balance; this may also complement the observation that HAMA was not retained in the RigiScan model but was retained in the CDDU model. Future studies could explore fecal microbiome, short-chain fatty acids, serum Trp/kynurenine (Kyn)/5-HT, Tph2 expression or gene polymorphisms, and inflammatory factors as potential targets for stratification, efficacy monitoring, and intervention in sexual dysfunction, including ED and premature ejaculation.

In this study, LR was the most robust model in both internal holdout validation and 5 × 3 nested cross-validation, suggesting that in low-dimensional, small-sample settings, linear models may be more robust than ensemble algorithms, and that metabolic indicators may have an approximately linear association with failure to meet NPTR criteria. However, the AUC of all models did not exceed 0.80, reaching only moderate discrimination, indicating that a discriminant model based on limited routine hematological indicators cannot yet replace objective RigiScan testing. AUC alone cannot establish utility for screening or risk stratification; calibration, Brier score, PPV, NPV, and decision curve analysis are also needed for comprehensive assessment. This means that although glycemic and weight indicators provide important clues regarding associations, the current model still cannot explain a considerable proportion of the variation associated with failure to meet NPTR criteria. Therefore, in clinical practice, the above metabolic indicators may be used for preliminary risk stratification; however, when the model-predicted probability is high or there is strong clinical suspicion of organic etiology, RigiScan monitoring should still be recommended to assist diagnosis.

Compared with previous studies on metabolic indicators and ED, the incremental contributions of this study are: (1) objective RigiScan-based grouping; (2) training-set-only feature selection and nested cross-validation; (3) SHAP-based model interpretation; and (4) systematic comparison with prior CDDU-based models. The association between metabolic indicators and ED has been widely reported; the value of this study lies in methodological rigor and interpretability rather than in discovering new risk factors.

This study has several limitations. First, this was a single-center, retrospective, cross-sectional study without external independent validation; the results only suggest associations between variables and cannot establish causality, and the related mechanistic hypotheses still require confirmation by prospective, multicenter studies. The exclusion of patients with neurogenic ED and a history of pelvic surgery may limit the generalizability of our findings to the broader organic ED population. Second, all models were trained with fixed default hyperparameters and were not specifically tuned; therefore, the AUC of each model may not represent its optimal performance. Third, although RigiScan monitoring was regarded as the reference standard, a small number of patients may still have been misclassified because of sleep quality or environmental factors. Sleep quality was not systematically assessed, and data on medications that may affect sleep or erection (e.g., benzodiazepines, antidepressants, PDE5 inhibitors, antihypertensives, 5α-reductase inhibitors) and washout periods were not collected; these factors may have confounded the NPTR results. Fourth, diabetes history and glucose-lowering treatment, ED duration, specific IIEF-5 scores, and relevant medication exposures were not systematically collected, which limits in-depth analysis of the dose–response relationship between metabolic indicators and NPTR outcomes. Fifth, although the three-way intersection strategy used for feature selection enhanced robustness, it may have excluded variables with potential clinical relevance, such as testosterone and age; future studies could explore customized models based on subgroup analyses or interaction terms. Sixth, this study did not include GBA-related indicators such as gut microbiota, short-chain fatty acids, and Trp/Kyn/5-HT, nor did it systematically integrate endothelial function, inflammatory, and oxidative stress markers; future studies could combine multi-omics strategies to further verify the mechanistic links among metabolic indicators, the GBA pathway, and failure to meet NPTR criteria. A sensitivity analysis comparing patients with one versus two valid monitoring nights was not performed, which may limit the robustness of the findings.

In summary, this study, based on objective RigiScan-based phenotypic classification and machine learning, revealed significant associations of BMI, GLU, HbA1c, Smoke, Hypertension, and Age with failure to meet the NPTR efficacy criterion. The LR model was the most robust in both internal holdout validation and nested cross-validation, but its discriminative power remained moderate and cannot yet replace RigiScan monitoring. Through systematic comparison with previous CDDU-based models, it further clarified the differences in the pathophysiological dimensions captured by the two examination methods and their complementary clinical value. These findings provide a quantitative reference for using simple metabolic indicators to estimate the probability of failure to meet NPTR criteria and implement stratified management in clinical settings where RigiScan is not yet widely available, and they also provide data-driven clues for future research integrating glycemic control and weight management into comprehensive ED intervention strategies.

## 5 Conclusion

Based on objective RigiScan grouping, machine learning models successfully identified metabolic factors closely associated with failure to meet the NPTR efficacy criterion, including BMI, GLU, HbA1c, Smoke, Hypertension, and Age. Clinically, glycemic control and weight management may be considered in the comprehensive care of patients with ED. The LR model was the most robust in both internal validation and nested cross-validation, but its discriminative power remained moderate and cannot yet replace RigiScan monitoring. These findings provide a data-driven reference for the etiological screening and stratified management of failure to meet NPTR criteria, although causal inferences cannot be drawn from this cross-sectional study.

## Data Availability

The original contributions presented in the study are included in the article/**Supplementary Material**. Further inquiries can be directed to the corresponding authors.

## Glossary

ALT: alanine aminotransferase
AST: aspartate aminotransferase
AUC: area under the receiver operating characteristic curve
BMI: body mass index
BUN: blood urea nitrogen
CDDU: color Doppler duplex ultrasonography
cGMP: cyclic guanosine monophosphate
CI: confidence interval
Cr: creatinine
DBP: diastolic blood pressure
E2: estradiol
ED: erectile dysfunction
EDV: end-diastolic velocity
eNOS: endothelial nitric oxide synthase
FSH: follicle-stimulating hormone
GBA: gut-brain axis
G-mean: geometric mean
GLU: fasting blood glucose
HAMA: Hamilton Anxiety Rating Scale
HAMD: Hamilton Depression Rating Scale
HbA1c: glycated hemoglobin
HDL-C: high-density lipoprotein cholesterol
ICI: intracavernosal injection
IGF-1: insulin-like growth factor-1
Kyn: kynurenine
LASSO: least absolute shrinkage and selection operator
LDL-C: low-density lipoprotein cholesterol
LH: luteinizing hormone
LightGBM: light gradient boosting machine
LR: logistic regression
NEUT: neutrophil count
NO: nitric oxide
NPTR: nocturnal penile tumescence and rigidity
PRL: prolactin
PSV: peak systolic velocity
RF: random forest
RI: resistance index
RhoA: Ras homolog family member A
ROCK: Rho-associated coiled-coil containing protein kinase
ROC: receiver operating characteristic
SBP: systolic blood pressure
SHAP: Shapley additive explanations
SVM: support vector machine
T: total testosterone
TC: total cholesterol
TG: triglycerides
TNF-α: tumor necrosis factor-alpha
Trp: tryptophan
Tph2: tryptophan hydroxylase 2
5-HT: 5-hydroxytryptamine
UA: uric acid
XGBoost: extreme gradient boosting machine.
