# Supplementary material for: Identification of factors associated with failure to meet nocturnal penile tumescence and rigidity criteria based on RigiScan monitoring and machine learning algorithms: a cross-sectional study
Source: Front Endocrinol (Lausanne). 2026 Oct 27;17:1926525. doi: 10.3389/fendo.2026.1926525 (PMC13747787; doi:10.3389/fendo.2026.1926525)
Supplement: Supplementary file 1 [file SupplementaryFile1.docx]

**Reference intervals for laboratory parameters**

ALT：9~50 U/L

AST：15~40 U/L

ALB：40~55 g/L

BUN：3.10~8.00 mmol/L

Cr：57.0~97.0 μmol/L

UA：202.30~416.50 μmol/L

CysC：0.51~1.09 mg/L

GLU：4.11~5.89 mmol/L

TC：<5.20 mmol/L

TG：<2.26 mmol/L

HDL-C：>1.45 mmol/L

LDL-C：2.59~3.34 mmol/L

T：248.00~835.00 ng/dL

E2：11~43 ng/dL

LH：1.24~8.62 mIU/mL

FSH：1.5~12.4 mIU/mL

PRL：4.00~15.20 ng/dL

IGF-1：60~350 ng/mL

Hb：130~175 g/L

PLT：125~350 ×10⁹/L

NEUT：1.80~6.30 ×10⁹/L

HbA1c：4.0~6.0 %
